# Mice lacking RAP1 show early onset and higher rates of DEN-induced hepatocellular carcinomas in female mice

**DOI:** 10.1371/journal.pone.0204909

**Published:** 2018-10-11

**Authors:** Iole Ferrara-Romeo, Paula Martínez, Maria A. Blasco

**Affiliations:** Telomeres and Telomerase Group, Molecular Oncology Program, Spanish National Cancer Centre (CNIO), Melchor Fernández Almagro 3, Madrid, Spain; Tulane University Health Sciences Center, UNITED STATES

## Abstract

RAP1, a component of the telomere-protective shelterin complex, has been shown to have both telomeric and non-telomeric roles. In the liver, RAP1 is involved in the regulation of metabolic transcriptional programs. RAP1-deficient mice develop obesity and hepatic steatosis, these phenotypes being more severe in females than in males. As hepatic steatosis and obesity have been related to increased liver cancer in mice and humans, we set out to address whether RAP1 deficiency resulted in increased liver cancer upon chemical liver carcinogenesis. We found that *Rap1*^*-/-*^ females were more susceptible to DEN-induced liver damage and hepatocellular carcinoma (HCC). DEN-treated *Rap1*^*-/-*^ female livers showed an earlier onset of both premalignant and malignant liver lesions, which were characterized by increased abundance of γH2AX-positive cells, increased proliferation and shorter telomeres. These findings highlight an important role for RAP1 in protection from liver damage and liver cancer.

## Introduction

Primary liver cancer is the fifth and seventh most common cancer in men and women, respectively and the second leading cause of cancer-related death worldwide [1]. Hepatocellular carcinoma (HCC) represents approximately 90% of all cases of primary liver cancer [1]. The main risk factors for HCC development include viral hepatitis, alcohol-induced hepatitis and non-alcoholic steatohepatitis (NASH) [2]. The incidence of liver cancer is increasingly on the rise and this is at least partly due to the growing epidemics of obesity and metabolic syndrome [3].

Mammalian telomeres are formed by TTAGGG repeats bound by a six-protein complex known as shelterin, which ensures telomere protection. The shelterin complex is composed of six core proteins, TRF1, TRF2, TIN2, POT1, TPP1 and RAP1 (for a review see [4–15]. Telomeres shorten with each cell division owing to the so-called “end-replication problem” [16, 17]. Telomerase activity can compensate for telomere shortening by the addition of de novo TTAGGG repeats onto chromosome ends [18]. Telomerase is formed by a catalytic subunit known as TERT and an associated RNA component or Terc that is used as template for the addition of new telomeric repeats [18]. Telomerase is expressed in pluripotent stem cells; however, it is downregulated after birth in the majority of somatic tissues, contributing to telomere shortening with aging [19]. Loss-of-function mutations in *hTERT* and *hTERC* are associated with familial liver diseases marked by fibrosis and inflammation [20, 21]. In addition, telomere shortening has been shown to represent a causal factor impairing liver regeneration and accelerating cirrhosis, a main risk factor for liver cancer development [22]. Indeed, telomere shortening has been associated with cancer development in the liver [23–25]. Interestingly, alterations in the expression of shelterin coding genes have also been identified in cirrhosis and HCC, suggesting that the development of HCC involves the dysregulation of telomere protective factors [26]. Mouse models deficient for shelterin genes have suggested a role of shelterin proteins in HCC development [27, 28]. In particular, mice deficient for TRF1 in the liver, develop large liver cell changes (LLCC) frequently found in liver cirrhosis in response to chronic replicative stress [28]. Similarly, transient depletion of the shelterin TRF2 in hepatocytes results in increased liver cancer [27]. These findings suggested that dysfunctional telomeres can induce DNA damage and telomere aberrations in the liver, which in the eventual loss of tumor suppressor genes such as p53, could lead to increased tumorigenesis [29].

In addition, the telomerase gene has been also found mutated in human HCC. In particular, whole-exome sequencing found a mutation hotspot in the telomerase (*TERT*) promoter, as well as TERT focal amplification [30, 31]. Interestingly, TERT promoter mutations were found at early HCC stages, pinpointing TERT as a key player in hepatocarcinogenesis by allowing the immortalization of neoplastic clones [30]. Telomerase deficient mice are protected from HCC development but present a significant increase of early stages neoplastic lesions as compared to wild-type mice, indicating that telomere biology exerts a dual role in the initiation and progression of HCC [32–34].

RAP1 binds to telomeric repeats through its interaction with TRF2 [5, 35, 36]. Mouse RAP1 is not a key factor for telomere maintenance and protection in the presence of sufficient telomere reserve but plays a crucial role in the context of telomerase deficiency [37–39]. RAP1 can also bind throughout the chromosome arms where it regulates gene expression [37, 38, 40–42]. Another non-telomeric function for RAP1 was revealed in the cytoplasm, where it acts as a modulator of the NF-kB signaling pathway by interacting with IKK complex. The RAP1-IKK interaction is required for the phosphorylation of the p65 subunit of NF-kB, enabling it to perform gene transcriptional activation [43]. RAP1-deficient mice do not have severe telomere phenotypes and can live to adulthood; however, they develop hepatosteatosis and are prone to obesity, being these phenotypes more severe in females than in males [40, 42]. Gene expression profile analyses in liver of adult mice revealed that in the absence of RAP1 several metabolic pathways including fatty acid metabolism, PPARα signalling and glucose metabolism are remarkably affected [40]. PPARα (Peroxisome proliferator-activated receptor alpha) is a ligand-activated transcription factor that belongs to the nuclear hormone receptor superfamily and is a major regulator of hepatic energy control [44, 45]. Together with its cofactor PGC1α (PPARγ Co-activator 1α), PPARα regulates the expression of genes involved in fatty acid beta-oxidation, lipid metabolism, gluconeogenesis, inflammation, atherosclerosis and autophagy [44–46]. In the absence of RAP1, PPARα and PGC1α levels are decreased leading to deregulation of several of their target genes and the subsequent deregulation of metabolic pathways involved in hepatic energy homeostasis. These molecular alterations are concomitant to increased incidence of obesity, which similarly to that described for Pparα- and Pgc1α-deficient mice [47–51], are more pronounced in RAP1-deficient females [40]. Of note, despite the effect of RAP1-deficiency in obesity and on hepatosteatosis development, RAP1-deficient mice do not spontaneously develop liver cancer. Indeed, no difference in tumor incidence has been reported between *Rap1*^+/+^ and *Rap1*^-/-^ mice, in both genders [40]. The role of the PPARα signalling in liver carcinogenesis is unclear. On one hand, PPARα-deficiency was shown to enhance the susceptibility to DEN initiated HCC [52]. On the othe hand, loss of PGC1α was shown to protect against DEN-induced liver cancer [53]. This role of RAP1 in regulating important metabolic pathways in the liver [40, 42], may suggest a role for RAP1 in liver cancer.

Here we analyze the role of RAP1 in DEN-induced carcinogenesis using both male and female *Rap1*^*-/-*^ mice. Similar to humans, a very pronounced gender disparity is observed in mouse HCC models, males being more prone to develop HCC than females [54, 55]. We found that *Rap1*^*-/-*^ female mice were more susceptible to DEN-induced HCC than wild-type controls as indicated by earlier onset and increased number of both pre-neoplastic lesions and HCC, which was accompanied by a significantly decreased lifespan as the consequence of liver cancer. Before humane end-point, DEN-induced female HCC lacking RAP1 showed increased abundance of γH2AX, AC3 and Ki67 positive cells as well as shorter telomeres as compared to wild-type control HCC, reflecting the higher proliferative history of RAP1-deficient tumors.

## Results

### DEN-induced liver damage hampers body weight gain as a consequence of RAP1 deficiency

**Fig 1:**
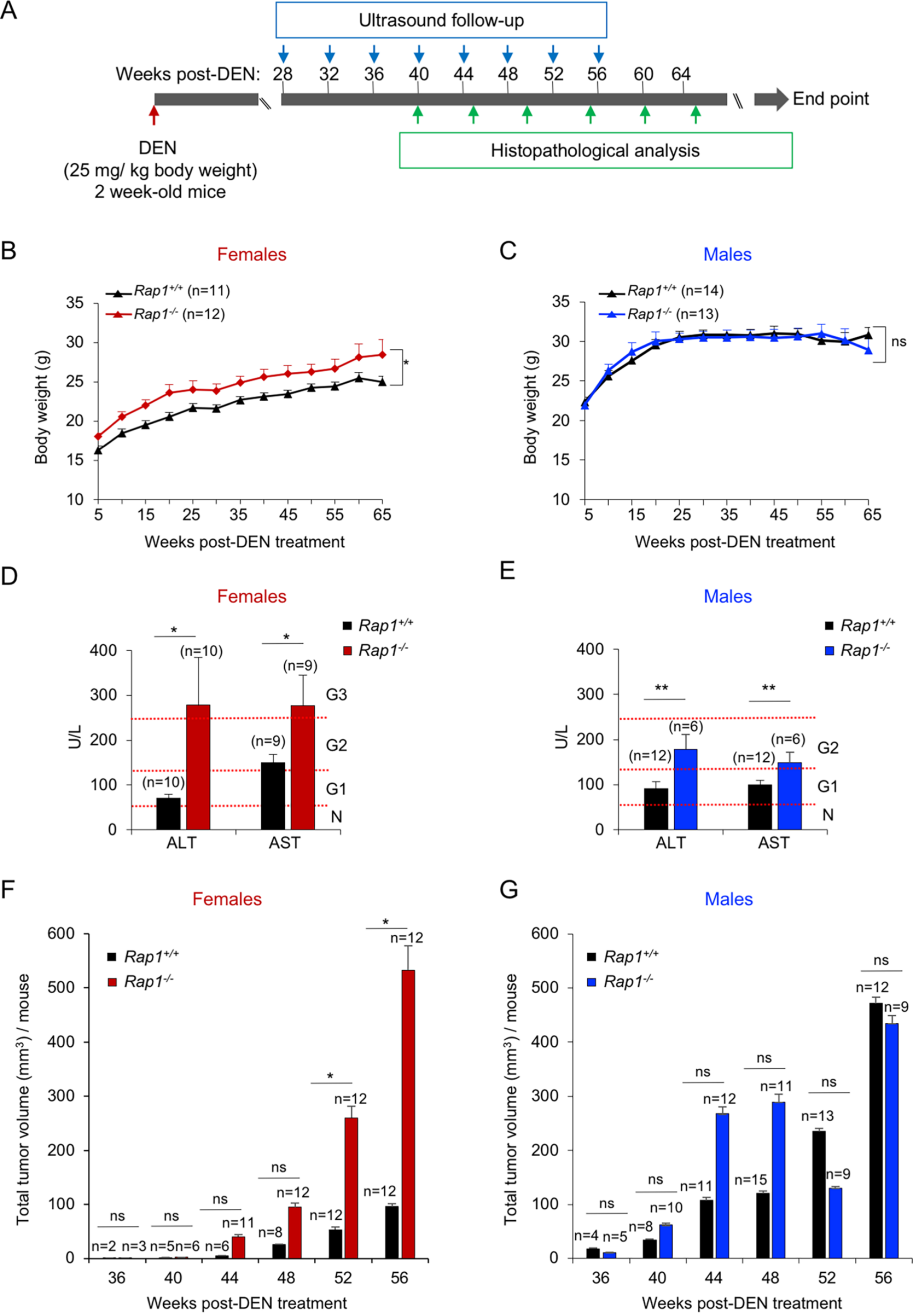
RAP1 deficiency leads to a higher susceptibility to DEN-induced liver damage and HCC development. (**A**) Two-week-old *Rap1*^*+/+*^ and *Rap1*^*-/-*^ female and male mice were intraperitoneally injected with DEN (25mg/kg body). Liver lesions were longitudinally monitored by ultrasound analysis from 28 weeks onwards after DEN treatment every fourth week. A group of *Rap1*^*+/+*^ and *Rap1*^*-/-*^ females were sacrificed at 40-, 45-, 55-, 60- and 65 weeks post-DEN for histopathological analysis. The rest of the mice were sacrificed at humane endpoint. (**B-C**) Body weight gain of *Rap1*^*+/+*^ and *Rap1*^*-/-*^ females (B) and males (C) from the fifth week post-DEN onward. (**D-E**) Plasma levels analysis of alanine (ALT) and aspartate (AST) aminotransferases in *Rap1*^*+/+*^ and *Rap1*^*-/-*^ females at 50–60 weeks post-DEN (D) and males at 50–55 weeks post-DEN (E). Females at 50- and 60- weeks post-DEN and males at 50- and 55- weeks post-DEN were grouped since no significant differences were observed between these two ages. For ALT analysis, 5 *Rap1*^*+/+*^ and 6 *Rap1*^*-/-*^ females at 50 weeks post-DEN and 5 *Rap1*^*+/+*^ and 4 *Rap1*^*-/-*^ females at 60 weeks post-DEN were analyzed. For AST analysis, 4 *Rap1*^*+/+*^ and 5 *Rap1*^*-/-*^ females at 50 weeks post-DEN and 5 *Rap1*^*+/+*^ and 4 *Rap1*^*-/-*^ females at 60 weeks post-DEN were analyzed. For ALT and AST analysis in males, 4 *Rap1*^*+/+*^ and 2 *Rap1*^*-/-*^ mice at 50 weeks post-DEN and 8 *Rap1*^*+/+*^ and 2 *Rap1*^*-/-*^ mice at 55 weeks post-DEN were analyzed. Hepatotoxicity grades are shown to the right. N, normal; G1, grade 1; G2, grade 2 and G3, grade 3. G1 hepatotoxicity was defined as a serum ALT level of 51–125 U/L, G2 as a serum ALT level of 126–250 U/L and G3 as a serum ALT level of 251–500 U/L. (**F-G**) Quantification of total volume of hepatic lesion by ultrasound between 36- and 56-weeks post-DEN in *Rap1*^*+/+*^ and *Rap1*^*-/-*^ females (F) and males (G). Values and error bars represent the mean and SE, respectively. N, number of mice. Statistical significance was determined by Student’s t test. *p<0.05, **p<0.01, ***p<0.001; ns, not significant.

To investigate a potential role of RAP1 in protection from hepatocarcinogenesis, we induced hepatocellular carcinoma (HCC) in both *Rap1*^*-/-*^ and wild-type mice by intraperitoneal administration of diethylnitrosamine (DEN) [40, 56]. DEN is a DNA alkylating agent that, by inducing DNA damage in the liver, eventually results in dysplastic foci (i.e. group of small dysplastic hepatocytes with an increased nuclear/cytoplasmic ratio), which can progress to multifocal HCC [57]. DEN was chosen at a concentration (25 mg /kg body weight) known to act as a complete carcinogen if injected into 2-week-old mice when hepatocytes are still actively proliferating [58, 59]. To address whether liver dysfunction associated to RAP1 deficiency synergizes with DEN-induced liver lesions in HCC development, 2- week-old *Rap1*^*-/-*^ and *Rap1*^*+/+*^ male and female mice were injected with DEN (25 mg /kg body weight). The onset and progression of liver lesions was monitored longitudinally by ultrasound analysis every fourth week from 28 weeks onwards after DEN treatment (Fig 1A). Groups of female mice from each genotype were sacrificed at 40-, 45-, 55-, 60- and 65- weeks post-DEN treatment, for histopathological analysis at shorter time-points. The rest of the mice were sacrificed when the presence of massive hepatic tumors was recognized as humane endpoint (Fig 1A).

To study the effects of DEN treatment on the previously described obesity phenotype of RAP1-deficient mice, we followed body weight longitudinally in the different mouse cohorts (Fig 1B and 1C). RAP1-deficient females treated with DEN showed a 10% increase in body weight compared to the previously reported 30% increase in body weight in the case of untreated *Rap1*^*-/-*^ females (Fig 1B)[40, 42]. No differences in body weight were observed in the case of the DEN-treated males (Fig 1C). In order to determine liver damage in the DEN treated mice, we analyzed the plasma levels of alanine (ALT) and of aspartate (AST) aminotransferases in males at 50 and 55 weeks post-DEN treatment and females at 50 and 60 weeks post-DEN treatment (Fig 1D and 1E). We found that both male and female *Rap1*-deficient mice showed significantly increased levels of ALT and AST compared to wild-type mice, indicating increased liver damage in the absence of RAP1 (Fig 1D and 1E). Indeed, the ALT and AST levels present in wild-type correspond to a grade 1 (51–125 U/L) while in RAP1-deficient males and females to grade 2 (126–250 U/L) and 3 (251–500 U/L) of hepatotoxicity, respectively [60].

Longitudinal ultrasound analysis of liver lesions showed a progressive increase in the total volume of liver lesions per mouse in DEN-treated males and females (Fig 1F and 1G). Indeed, RAP1-deficient females showed significantly larger liver lesions compared to wild-type females (Fig 1F), while the differences between RAP1-deficient and wild-type males did not reach statistical significance (Fig 1G). Male mice presented an earlier onset and a more rapid tumor growth than females, indicating a higher susceptibility of male mice for DEN-induced HCC as already reported [54]. Interestingly, *Rap1*^*-/-*^ females show a similar tumor volume as male mice from week 52 onwards, in contrast to wild-type females that present significantly lower tumor volume. These findings show an increased susceptibility of RAP1-deficient female mice to DEN-induced liver carcinogenesis, in agreement with *Rap1*^*-/-*^ females showing higher body weight and higher hepatotoxicity in response to DEN than wild-type controls.

**Fig 2:**
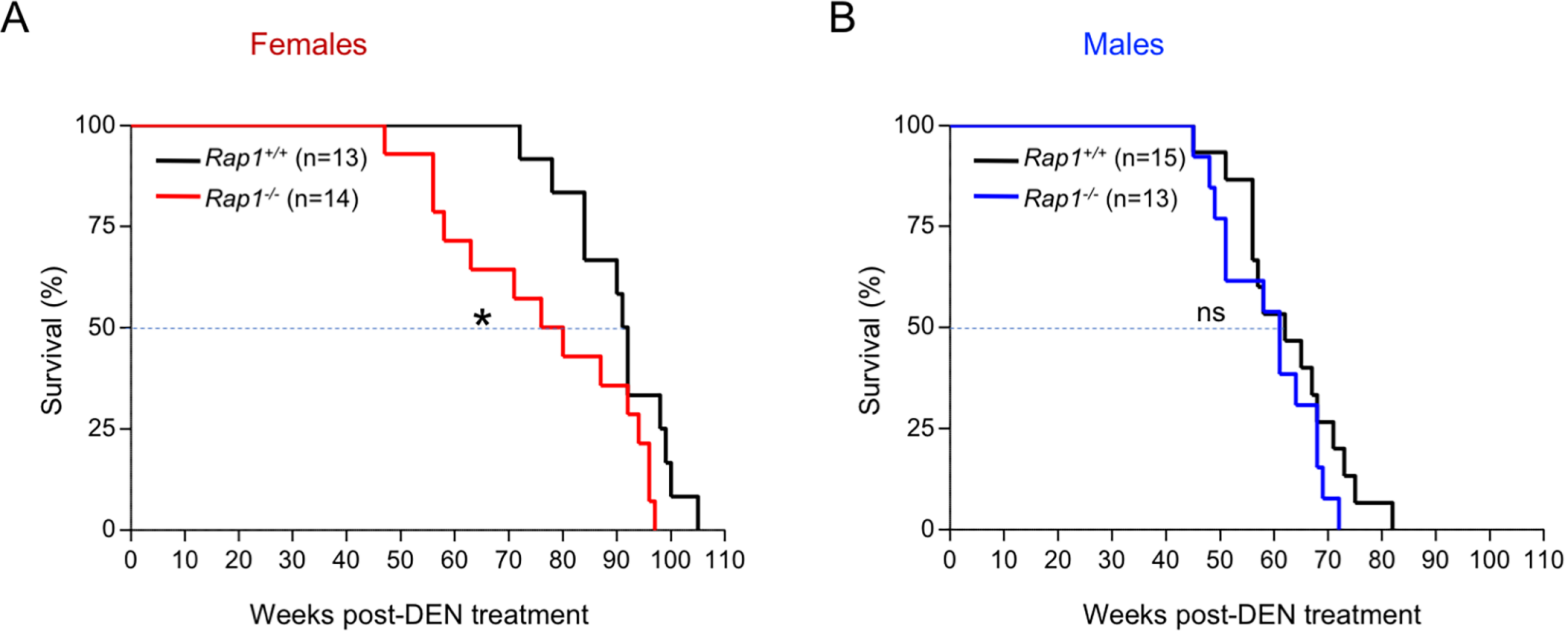
RAP1 deficiency leads to a reduced lifespan of DEN-treated female mice. (**A-B**) Kaplan-Meier survival curves of *Rap1*^*+/+*^ and *Rap1*^*-/-*^ female (A) and male (B) mice. Statistical significance was determined by the log rank test. *, p ≤ 0.05.); ns, not significant.

Finally, we followed the survival of the different mouse cohorts that died as a consequence of liver tumors (Fig 2 A and 2B). RAP1-deficient females presented a 15% decrease in median survival compared with wild-type females (78 and 91.5 weeks post-DEN, respectively) (Fig 2A). In contrast, no differences in survival in the male cohorts were observed, being the median survival of the *Rap1*^*+/+*^ and *Rap1*^*-/-*^ male mice of 62 and 61 weeks post-DEN, respectively (Fig 2B). The decreased survival of DEN-treated RAP1-deficient females as compared to wild-type controls may be explained by their increased liver damage and more rapidly growing liver lesions as detected by ultrasounds in response to the carcinogenic treatment.

### RAP1 deficient females are more susceptible to DEN-induced HCC than wild-type mice

**Fig 3:**
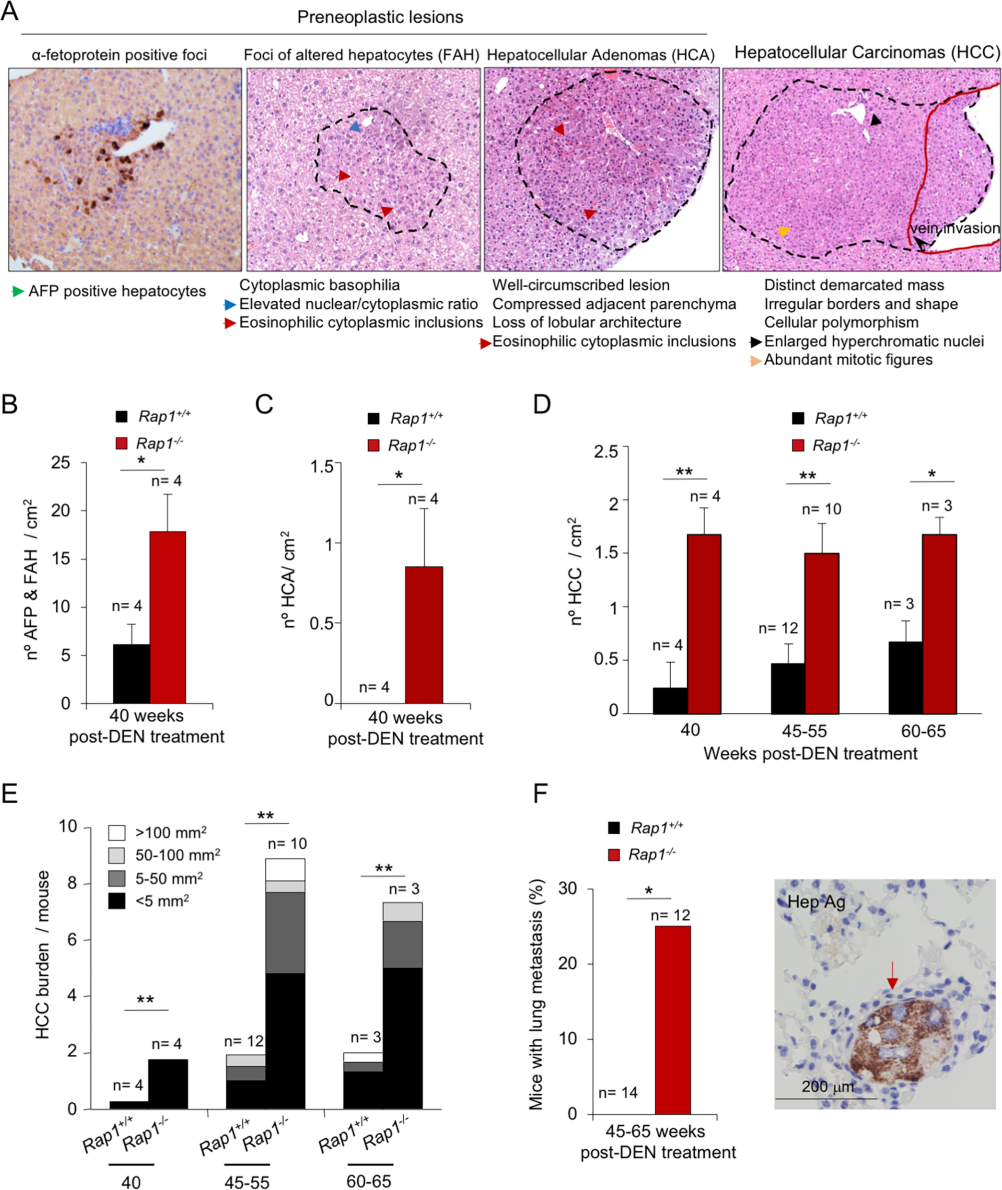
Earlier onset of premalignant and malignant hepatic lesions in DEN-treated *Rap1*^*-/-*^ females. **(A**) Representative light microscopy images of α-fetoprotein (AFP) and hematoxylin-eosin (H&E) liver sections showing the different types of lesions induced by DEN. Focus of AFP-positive hepatocytes, focus of altered hepatocytes (FAH), hepatocellular adenoma (HCA) and hepatocellular carcinoma (HCC) are shown from left to right. For a detailed histological description see Material and Methods. (**B-C**) Quantification of FAHs and AFP-positive foci (**B**) and HCAs (**C**) in *Rap1*^*+/+*^ and *Rap1*^*-/-*^ livers of female mice at 40 weeks after DEN injection. (**D**) Quantification of total number of HCC in *Rap1*^*+/+*^ and *Rap1*^*-/-*^ livers of female mice at 40, 45–55 and 60–65 weeks post-DEN. (**E**) Quantification of the number of HCC of different size in the mice described in (D). (**F**) Incidence of lung metastasis in *Rap1*^*+/+*^ and *Rap1*^*-/-*^ female mice between 45 and 65 weeks post-DEN. A representative light microscopy image of hepatocyte antigen-stained lung sections is shown to the right. Values and error bars represent the mean and SE, respectively. N, number of mice. Statistical significance was determined by Student’s t test.

To further analyze the tumor-prone phenotype of RAP1-deficient females in response to DEN, we sacrificed a group of *Rap1*^*-/-*^ and *Rap1*^*+/+*^ females at 40, 45–55 and 60–65 weeks post-DEN treatment and performed full histopathological analysis of the livers. The lesions were categorized as preneoplastic lesions and neoplastic lesions (Fig 3A). Preneoplastic lesions included α-fetoprotein (AFP) positive foci, foci of altered hepatocytes (FAH), and hepatocellular adenomas (HCA) [61, 62] (see Material and Methods). All the neoplastic lesions were HCC. We found that 40-weeks post-DEN treatment, the number of preneoplastic lesions including AFP & FAH as well as HCA was significantly higher in RAP1-deficient females compared to similarly treated wild-type females (Fig 3B and 3C). Interestingly, we also found a significantly increased number of HCC per cm^2^ of liver area in *Rap1*^*-/-*^ females compared to *Rap1*^*+/+*^ controls from 40 to 65 weeks post-DEN treatment (Fig 3D). Furthermore, the HCC burden (total number of lesions of different size per mouse) in the entire liver was significantly higher in *Rap1*^*-/-*^ compared to *Rap1*^*+/+*^ females from 40 to 65 weeks post-DEN treatment (Fig 3E).

HCC can metastasize to the lungs [63]. We therefore set to quantify the number of lung metastasis, by using an immunohistochemistry staining with the hepatocyte antigen (hep) in lung sections at 45–65 weeks post DEN treatment (Fig 3F). We found that 25% of RAP1-deficient females present clusters of hep-positive cells in the lungs while none of the wild-type females showed these lesions, indicating that RAP1 absence speeds up tumor spreading to the lungs (Fig 3F).

### Increased proliferation, DNA damage and apoptosis in HCC lacking RAP1

**Fig 4:**
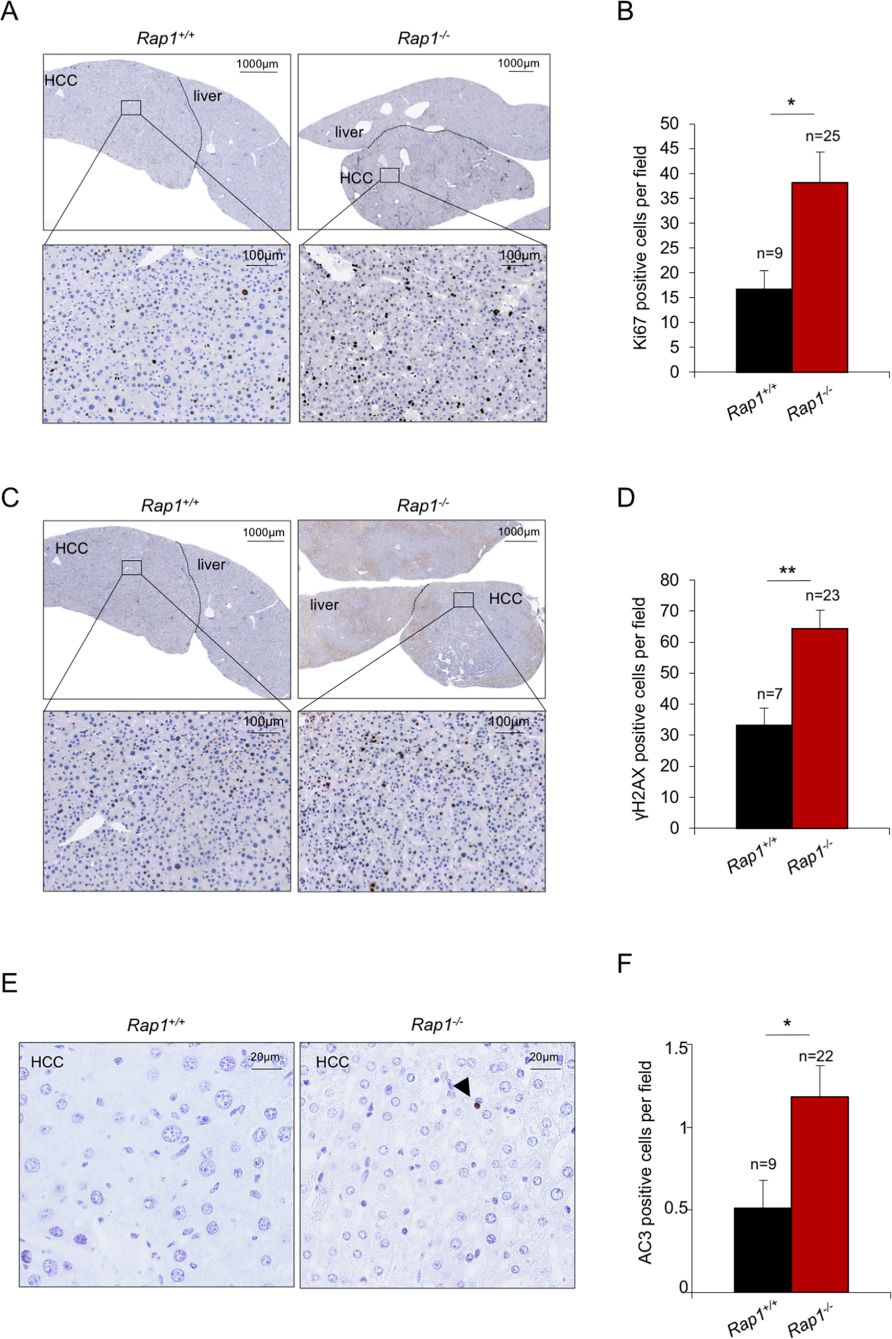
Increased proliferation, DNA damage and apoptosis in HCC lacking RAP1 in females. **(A-F**) Representative images and quantification of Ki67 (A, B) γH2AX (C-D) and AC3-positive cells **(**E-F) in *Rap1*^*+/+*^ and *Rap1*^*-/-*^ tumor sections of female mice at 50–55 weeks post-DEN treatment. Tumors from 6 *Rap1*^*+/+*^ and 6 *Rap1*^*-/-*^ females were analyzed. Three females from each cohort were at 50- and three at 55- weeks post-DEN. Mice of 50 and 55 weeks post-DEN were grouped since no significant differences were observed between these two ages. The size of the analyzed tumors ranged from 5 to 20 mm^2^. Values and error bars represent the mean and SE, respectively. N, number of tumors. Statistical significance was determined by Student’s t test. *p<0.05, **p<0.01, ***p<0.001.

In order to address the molecular events underlying the increased neoplastic lesions in RAP1-deficient females in response to DEN treatment, we set to analyze different molecular markers in HCC at 50–55 weeks post-DEN treatment before humane end point. In particular, we measured proliferation (Ki67-positive cells), DNA damage (γH2AX-positive cells) and apoptosis (AC3-positive cells) by immunohistochemistry in tumors from both wild-type and RAP1-deficient females (Fig 4A–4F). We found that RAP1-deficient HCC showed a 2-fold increase in Ki67-positive cells compared to wild-type HCC, in agreement with higher tumor growth rate in *Rap1*^*-/-*^ as compared to *Rap1*^*+/+*^ females (Figs 4A, 4B, 3D and 3E). RAP1-deficient HCC also presented increased cells with DNA damage (γH2AX-positive cells) and increased apoptosis compared to wild-type controls (Fig 4C–4F). The higher DNA damage burden in the RAP1-deficient HCC may reflect either the higher susceptibility of *Rap1*^*-/-*^ females to DEN-induced hepatocarcinogenesis or the more advanced stages of RAP1-deficient tumors as compared to wild-type controls at this time point.

### Role of RAP1 in HCC telomere dynamics

**Fig 5:**
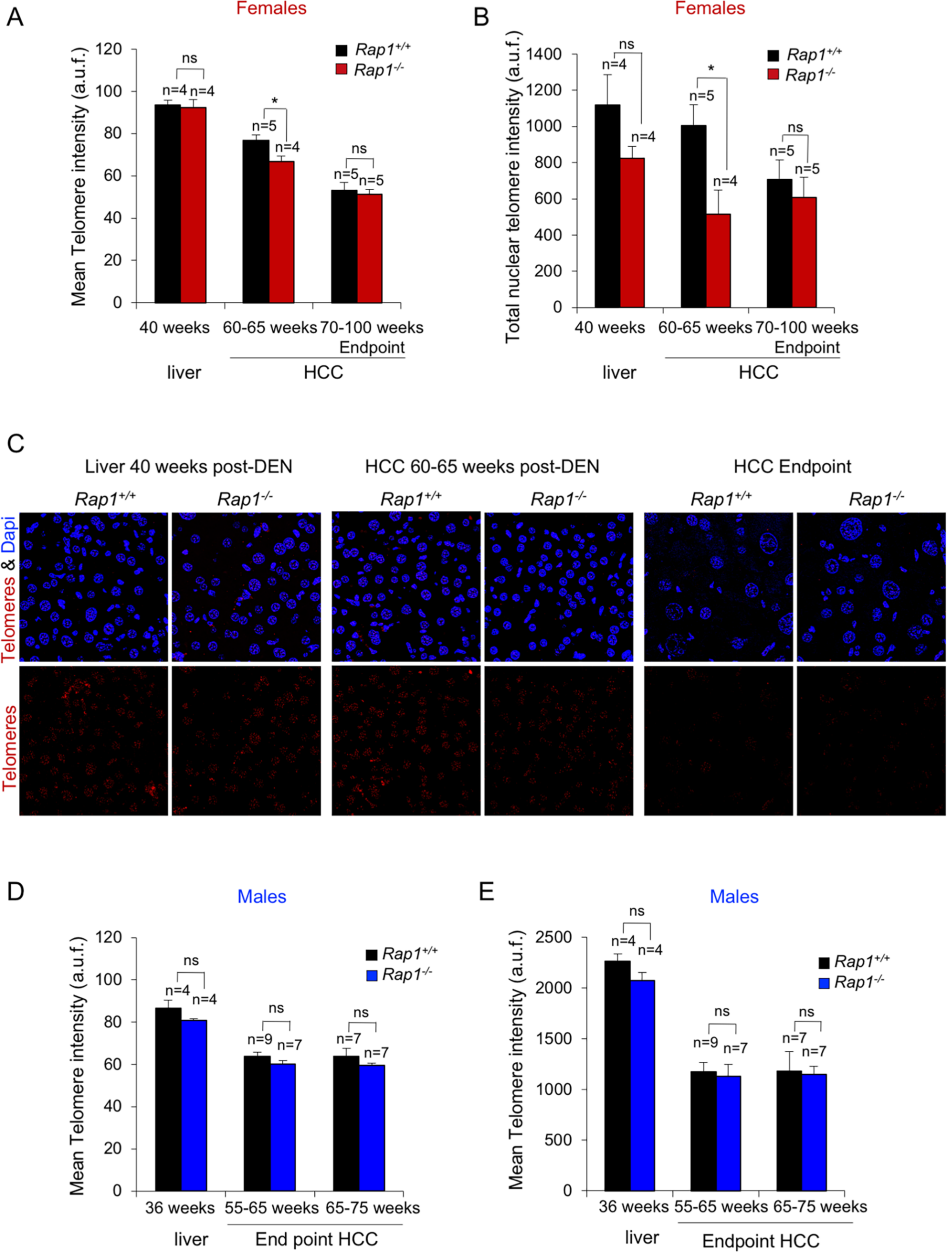
RAP1 deficiency does not affect telomere length in full blown HCC. **(A-B**) Mean telomere fluorescence (A) and total nuclear telomere fluorescence (B) in liver sections at 40 weeks post-DEN, 60–65 weeks post-DEN and at the endpoint at 70–100 weeks post-DEN. At 40 weeks the telomere intensity was determined in healthy hepatic tissue. At 60–65 weeks post-DEN before humane endpoint and at the humane endpoint, telomere intensity was determined in HCC. At 60–65 weeks post-DEN, the tumors analyzed presented an area between 10–30 mm^2^. At the endpoint, the analysis was performed in tumors with an area between 50–500 mm^2^. (**C**) Representative Q-fish images showing the telomere FISH (red) in *Rap1*^*+/+*^ and *Rap1*^*-/-*^ liver and tumor sections of female mice at different time points. **(D-E**) Mean telomere fluorescence (D) and total nuclear telomere fluorescence (E) in liver sections at 36 weeks post-DEN and at the humane endpoint of male mice that died of liver tumors at either 55–65 or 65–75 weeks post DEN. At 36 weeks the telomere intensity was determined in healthy hepatic tissue. At the humane endpoint, telomere intensity was determined in HCC. In male mice at humane end points ranging from 55–65 or from 65–75 weeks post-DEN, the tumors analyzed presented an area between 50–350 mm^2^ and 100–500 mm^2^, respectively. a.u.f., arbitrary units of fluorescence. Values and error bars represent the mean and SE, respectively. N, number of tumors. Statistical significance was determined by Student’s t test. *p<0.05, **p<0.01, ***p<0.001.

Telomerase promoter mutations in human HCC highlight the importance of telomere length maintenance for liver cancer [30, 31]. Here, we set to address whether RAP1-deficiency impacted on telomere length in the context of DEN-induced liver carcinogenesis. To this end, we analyzed telomere length in female liver sections at 40-weeks and in HCC at 60–65 weeks post-DEN treatment before end-point, and in HCC at the humane endpoint from females at 70–100 weeks post-DEN (Fig 5A–5C). No significant differences were observed between *Rap1*^*-/-*^ and *Rap1*^*+/+*^ samples in either healthy liver tissue at 40 weeks or in HCC at the endpoint when tumors have reached maximal size between. In contrast, at 60–65 week post-DEN treatment before humane end-point, we found that RAP1-deficient HCC showed both decreased mean telomere intensity and decreased total nuclear telomere intensity (Fig 5A–5C).

We also analyzed telomere length in male liver sections at 36-weeks and in HCC at the humane endpoint from males that died at 55–65 or 65–75 weeks post-DEN (Fig 5D and 5E). No significant differences were observed between *Rap1*^*-/-*^ and *Rap1*^*+/+*^ samples in either healthy liver tissue at 36 weeks or in HCC at the endpoint when tumors have reached maximal size between (Fig 5D and 5E).

In accordance with previous work, these data confirm that RAP1 deficiency does not impact on telomere length in healthy liver tissue, neither in females nor in males [40]. These results also indicate that RAP1 deficiency does not have any effect in telomere length maintenance in full blown HCC at the end-point, suggesting that telomere length is neither a driver nor a limiting factor for tumor development and progression in these settings. In addition, female and male HCCs present shorter telomeres as compared to cells in healthy liver tissue indicating telomere shortening associated to malignant cell proliferation (Fig 5A–5E). The observation that female HCCs at week 60–65 post-DEN before humane endpoint present shorter telomeres as compared to wild type HCCs, probably reflects the proliferative history and more advanced stages of *Rap1*^*-/-*^ tumors as compared to *Rap1*^*+/+*^ controls (Fig 5A–5C). Indeed, a RAP1-independent progressive telomere shortening is observed when comparing HCCs before human endpoint to HCCs at human endpoint, both in *Rap1*^*+/+*^ and in *Rap1*^*-/-*^ female mice (Fig 5A–5C).

## Discussion

RAP1 is part of the shelterin complex that protects telomeres [36]. Murine RAP1 does not seem to be required for telomere capping and telomere length maintenance under normal conditions [37, 39, 64]. However, we recently showed that RAP1 is important for telomere length maintenance in the context of cellular stress conditions such as telomerase deficiency [64]. In addition, we previously showed that RAP1 can associate to non-telomeric genomic sites where it regulates gene expression [37, 38, 40–42]. RAP1-deficient mice are obese and develop signs of metabolic syndrome, including liver steatosis, which are more acute in females. [40, 42]. This is accompanied by altered gene expression profiles affecting several metabolic pathways [40]. As metabolic syndrome and obesity has been associated with increased liver cancer [2, 3, 65, 66], here we set to address whether RAP1 deficiency resulted in increased liver cancer in response to treatment with a widely used carcinogen, DEN. We found increased liver damage and increased tumor susceptibility of RAP1- deficient as compared to wild type female mice. Indeed, *Rap1*^*-/-*^ females showed increased number of both premalignant and malignant lesions and the tumors more rapidly reached larger sizes leading to significantly decreased Rap1-deficient female mouse survival upon DEN treatment. In addition, *Rap1*^*-/-*^ female HCC metastasized to the lung at earlier time-points than wild-type HCC, also indicating that *Rap1*^*-/-*^ females are more susceptible to DEN-induced hepatocyte malignization. These observations are in agreement with a more prominent effect of RAP1-deficiency in fat accumulation in female mice [40, 42].

Non- alcoholic fatty liver disease (NAFLD) is a well-known risk factor for HCC development [3, 65, 66]. NAFLD is characterized by increased hepatic lipid accumulation and diminished ability of the liver to metabolize several substrates. NAFLD progress to NASH, in which hepatocyte metabolic stress induces cell death, production of damage-associated molecules and chronic inflammation [65, 67]. Differentiated hepatocytes are able to re-enter the cell cycle and replace damaged cells and therefore the liver has the ability to repair itself after acute damage [68]. However, in chronic necro-inflammation, constant cell death, compensatory regeneration and activation of non-parenchymal cells, together with an altered immune response, promote liver fibrosis and tumorigenesis [65]. Necroinflammation also induces replicative stress, DNA damage and genetic instability that is detectable in preneoplastic lesions [69]. Our data indicate that the role of RAP1 in hepatocarcinogenesis is mediated by its function in metabolism regulation rather than to its telomeric function. Indeed, RAP1 has no effect in male hepatocarcinogenesis while it does in females in which RAP1 role in fat accumulation is predominant [40, 42]. Although a RAP1 telomeric function cannot be ruled out, our data underlines that the effect of RAP1 deficiency in NAFLD development synergizes with DEN-induced liver damage resulting in a faster HCC development in female mice lacking RAP1.

## Material and methods

### Mice generation and handling

All mice were generated and maintained at the Animal Facility of the Spanish National Cancer Research Centre (CNIO) under specific pathogen-free conditions in accordance with the recommendation of the Federation of European Laboratory Animal Science Associations (FELASA). *Rap1*^*+/+*^ and *Rap1*^*-/-*^ mice were generated by mating heterozygous (*Rap1*^*+/-*^) males and females [40]. Food (Harlan Laboratories) and water were provided *ad libitum* and measurements of the body weight were performed monthly. All animal experiments were approved by the Ethical Committee (CEIyBA) (IACUC.015-2014, CBA_21_2014) and performed in accordance with the guidelines stated in the International Guiding Principles for Biomedical Research Involving Animals, developed by the Council for International Organizations of Medical Sciences (CIOMS).

Mice included in the survival study were monitored two or three times per week. After the detection of the first tumor by ultrasounds, mice were monitored daily. We applied humane endpoint criteria and euthanized the animals when they showed signs of pain, sickness, suffering, or moribund conditions. In particular: loss of body weight, presence of big abdominal masses, reduced mobility, and hunched body posture were used as main criteria to recognize humane endpoint. When mice reached endpoint criteria, they were euthanized within a maximum of 24h. Euthanasia was performed in a CO2 gas chamber. Two of the twenty-six mice included in the survival study died unexpectedly before showing criteria for euthanasia. In both cases necropsy was performed and the presence of massive hepatic tumors was recognized as the cause of death.

### Tumor induction and serum analysis

Fourteen-day-old mice were injected intraperitoneally with 25 mg/kg of DEN (Sigma) [56]. Serum ALT and AST levels were determined using ABX Pentra (Horiba Medical).

### Ultrasound imaging and tumor quantification

Animals were anesthetized with Isofluorane (Isovet, Braun Vetcare) (4% during anesthetic induction and 2% as maintenance level) and livers were scanned (Vevo 770, 40 Mhz frequency) for structural alterations, including echogenicity variations, by using the probe RMV707b (Visualsonics, Canada). The frame rate used is in the range of 60 Hz and 11X11 mm FOV (field of view). The three diameters dimensions were measured (width, W, length, L, and depth, D) and the tumor volume was calculated by using the formula V = π/6xWxLxD [70].

### Hepatotoxicity grading

Criteria based on the World Health Organization Adverse Reaction Terminology (WHO-ART) were utilized to grade hepatotoxicity. Grade 1 hepatotoxicity was defined as a serum ALT level of 51–125 IU/L, or 1.25–2.5 times normal; grade 2 as a serum ALT level of 126–250 IU/L, or 2.6–5.0 times normal; grade 3 as a serum ALT level of 251–500 IU/L [60]

### Immunohistochemistry analysis

Tissue samples were fixed in 10% buffered formalin, dehydrated, embedded in paraffin wax and sectioned at 2.5 mm. Slides were deparaffinized in xylene and re-hydrated through a series of graded ethanol until water. Serial sections were stained with hematoxylin and eosin for pathological examination and lesions quantification. Serial paraffin sections of liver and lung samples were analyzed. The classification of the hepatic lesions was performed by a trained pathologist in a blinded manner.

Immunohistochemistry was performed on de-paraffined liver or lung sections processed with 10 mM sodium citrate (pH 6.5) cooked under pressure for 2 min. Slides were washed in water, then in Buffer TBS Tween20 0.5%, blocked with peroxidase, washed with TBS Tween20 0.5% again and blocked with fetal bovine serum followed by another wash. The liver slides were incubated with the primary antibodies: goat polyclonal to alpha-fetoprotein (R&D systems), mouse monoclonal to phospho-Histone H2AX (ser139) (JBW301, Millipore), rabbit monoclonal to Ki-67 (D3B5, Cell Signaling) or rabbit polyclonal to C3 cleaved-caspase 3 (Asp175) (Cell Signaling); the lung slides were incubated with the primary antibody: mouse monoclonal to human hepatocytes (OCH1E5, DAKO). Slides were then incubated with secondary antibodies conjugated with peroxidase from DAKO. Sections were lightly counterstained with hematoxylin and analyzed by light microscopy. Pictures were taken using Olympus AX70 microscope. The percentage of positive cells was quantified by eye.

Liver lesions were classified as follows. α-fetoprotein (AFP) positive foci are composed of hepatocytes positive for AFP with a cytosolic staining pattern. AFP is known to be expressed in early liver development and during hepatocarcinogenesis, but is largely absent in normal adult liver. AFP expression takes place during the early stages of hepatocarcinogenesis, before the appearance of histologically evident transformation, although not all HCC developed in mice are AFP positive [71]. Foci of altered hepatocytes (FAH) are composed of hepatocytes with increased cytoplasmic basophilia due to polyribosomes or rough endoplasmic reticulum, often smaller than normal, characterized by a high nuclear to cytoplasmic ratio that results in a “crowded” appearance. Eosinophilic cytoplasmic inclusions are frequently found within the hepatocytes in FAHs [61, 62]. Hepatocellular adenoma (HCA) are well-circumscribed lesions often causing compression of the adjacent parenchyma. The liver lobular architecture is not maintained, causing an irregular growth pattern that represents a primary distinction between HCA and FAH. Hepatocytes in HCA are well differentiated and variable in size, occasionally contain eosinophilic cytoplasmic inclusions. Degenerative changes such as lipidosis and cystic degeneration are frequently observed [61, 62]. Hepatocellular carcinoma (HCC) consist of demarcated masses with irregular borders due to the cellular invasion of the surrounding tissue. The lobular architecture is not maintained and the typical trabecular, glandular or solid growth patterns are observed. HCC are characterized by severe cellular polymorphism with enlarged and hyperchromatic nuclei and contain abundant mitotic figures. The masses are often characterized by presence of various size cysts, and hemorrhagic and/or necrotic areas [61, 62].

### Telomere length quantitative fluorescence (Q-Fish) analyses on liver sections

For quantitative telomere fluorescence *in situ* hybridization (Q-FISH), paraffin-embedded sections were deparaffinized and fixed with 4% formaldehyde, followed by digestion with pepsine/HCl and a second fixation with 4% formaldehyde. Slides were dehydrated with increasing concentrations of EtOH (70%, 90%, 100%) and incubated with the telomeric probe for 3 min at 85°C followed by 2h RT incubation in a wet chamber. In the final steps, the slides were extensively washed with 50% formamide and 0.08% TBS-Tween [72]. Confocal microscopy was performed at room temperature with a laser-scanning microscope (TSC SP5) using a Plan Apo 63Å-1.40 NA oil immersion objective (HCX). Maximal projection of z-stack images generated using advanced fluorescence software (LAS) were analyzed with Definiens XD software package. The DAPI images were used to detect telomeric signals inside each nucleus.

### Statistical analysis

The Kaplan-Meier method was used to estimate survival curves and log rank was used to evaluate statistical differences in median survival of the different mouse cohorts. A Student t-test was used to calculate the statistical significance (p) (p ≤ 0.05 = * and p ≤ 0.01 = **) in body weight, enzyme levels in serum, tumor burden, number of lesions, ki67, γH2AX and cleaved-caspase-3 expression. A chi-square test was used to calculate statistical differences in lung metastasis incidence.
